# Ankle ligament reconstruction after wide resection of the osteosarcoma of the distal fibula: a case report

**DOI:** 10.1186/s13104-017-3097-4

**Published:** 2017-12-28

**Authors:** Tanawat Vaseenon, Jirawat Saengsin, Amornrat Kaminta, Nuttaya Pattamapaspong, Jongkolnee Settakorn, Dumnoensun Pruksakorn

**Affiliations:** 1 Department of Orthopaedics, Faculty of Medicine, Chiang Mai University, 110 Intawaroros Road, Sriphum, Muang District, Chiang Mai, 50200 Thailand; 2 Department of Radiology, Faculty of Medicine, Chiang Mai University, 110 Intawaroros Road, Sriphum, Muang District, Chiang Mai, 50200 Thailand; 3 Department of Pathology, Faculty of Medicine, Chiang Mai University, 110 Intawaroros Road, Sriphum, Muang District, Chiang Mai, 50200 Thailand

**Keywords:** Distal fibulectomy, Fibula, Resection, Tendon transfer, Tumor

## Abstract

**Background:**

Restoration of the lateral ankle after distal fibulectomy is a difficult reconstructive procedure. Many surgical techniques have been proposed. This report shows another fibular reconstructive option with promising outcome.

**Case presentation:**

We report the case of a 30-year-old woman who presented with a solitary mass located in the lateral aspect of the ankle. The mass had grown rapidly for 2 months and caused increasing pain. Physical examination showed a 3.0 cm diameter tender, nonmobile hard mass in the lateral malleolus. Radiographs showed an osteolytic lesion involving the lateral cortex at the distal fibula. After incisional biopsy, pathologic examination found a well-differentiated intramedullary osteosarcoma. Neoadjuvant chemotherapy with doxorubicin was provided for 3 months prior to definitive surgical treatment. Magnetic resonance imaging showed persistent tumor in the biopsy site. After distal fibulectomy and wide resection, split tibialis posterior tendon transfer to the remaining peroneus brevis restored the stability of the ankle. The pain resolved within 3 months. The ankle was stable and no recurrence of the cancer was found at a 7 year follow-up.

**Conclusion:**

Reconstruction following distal fibulectomy and surrounding soft tissue resection responds favorably to split tibialis posterior transfer to the remaining peroneus brevis suggesting that this technique can provide a good and functional outcome.

**Electronic supplementary material:**

The online version of this article (10.1186/s13104-017-3097-4) contains supplementary material, which is available to authorized users.

## Background

Osteosarcoma is the most common non-hematologic primary bone tumor. The peak incidence of 44.77% is in the second decade of life and the most common site is the knee region [1]. Osteosarcoma of the fibula is rare, found in 2–5.6% of cases. It is especially rare at the distal fibula (approximately 0.47% of patients) [2]. Currently, the primary surgical treatments of distal fibular tumors involves local resection and reconstruction of the bone and the soft tissue. The goal of surgical resection of a malignancy is to achieve wide surgical margins. If wide surgical margins cannot be achieved due to anatomical constraints, a marginal resection combined with adjuvant or neoadjuvant treatments may be preferable to an amputation [3].

Distal fibulectomy, however, can lead to ankle joint instability and progressive valgus deformity [4]. Reconstruction of the bone and the soft tissue can be accomplished with procedures such as bone grafting, ligament and tendon augmentation, arthrodesis or prosthetic ankle joint replacement [2]. This study reports on the case of a 30-year-old woman with osteosarcoma of the distal fibula following wide resection and ankle ligament reconstruction.

## Case presentation

**Fig. 1:**
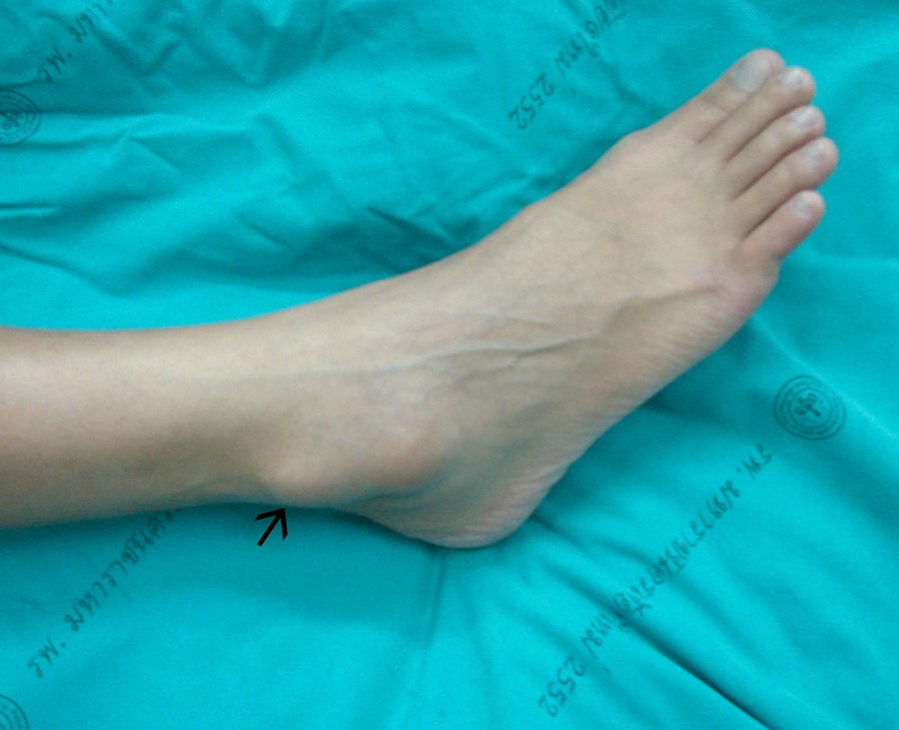
Preoperative clinical presentation of the tender, hard, fixed mass on the lateral aspect of the ankle

A 30-year-old Thai woman had had right ankle pain for 2 months and had a history of mild injury of the right ankle. Her ankle was swollen, especially on the lateral malleolus. Physical examination revealed a tender mass at the right lateral malleolus. Results of vascular, neurologic, and dermatologic examinations of the lower extremities were normal (Fig. 1).

**Fig. 2:**
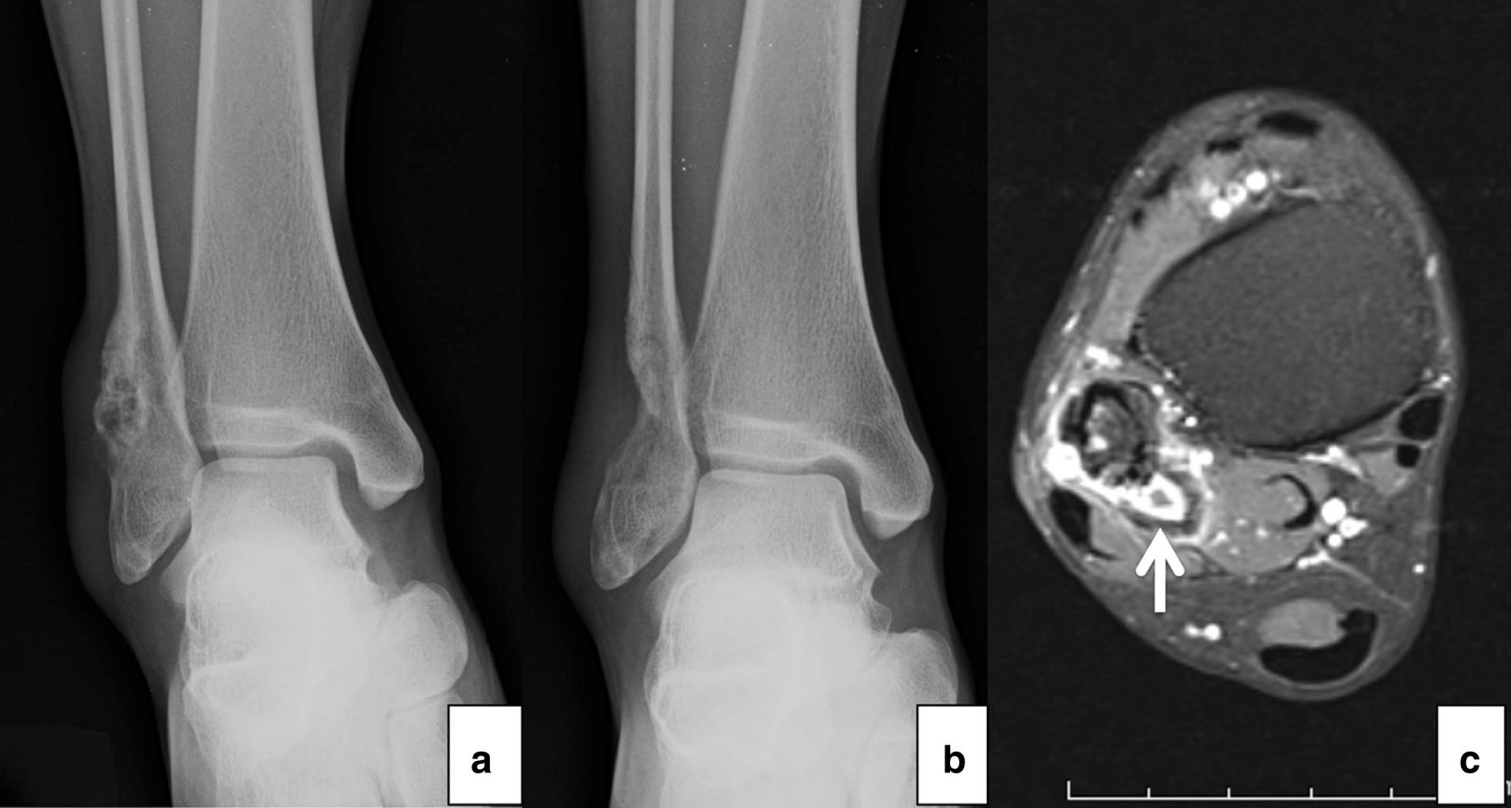
The initial radiograph showing an eccentric osteolytic lesion in the distal fibula (**a**) and post incisional biopsy (**b**). Axial magnetic resonance imaging (post intravenous gadolinium T1-weighted image with fat suppression) demonstrating a recurrent tumor near the biopsy site (arrow) (**c**)

Radiographs showed an eccentric osteolytic lesion in the distal fibula (Fig. 2a). An incisional biopsy was performed (Fig. 2b). Tissue pathology reported multiple pieces of white tan soft tissue and bone. Microscopic examination revealed numbers of cellular plump malignant cells with a high nuclear to cytoplasmic ratio, hyperchromatic nuclei, and prominent nucleoli. Mitotic figures were frequently observed (Fig. 4a). Neoplastic bone formation was noted (Fig. 4b, c). The tumor staging was Enneking stage IIB that were high grade in histology, tumor extension out of the bone without metastasis at initial diagnosis. Neoadjuvant chemotherapy with doxorubicin was administered for 3 months before definitive surgical treatment.

After chemotherapy, magnetic resonance imaging (MRI) was performed in order to evaluate the extension of disease and guide for definite tumor removal. The images revealed a 2.4 × 1.6 × 2 cm mass above the removed bone in the fibula, indicating a poor response to chemotherapy and persistent tumor. The tumor involved underneath the peroneus longus and brevis tendons (Fig. 2c). A CT scan of the lungs showed no pulmonary metastasis.

**Fig. 3:**
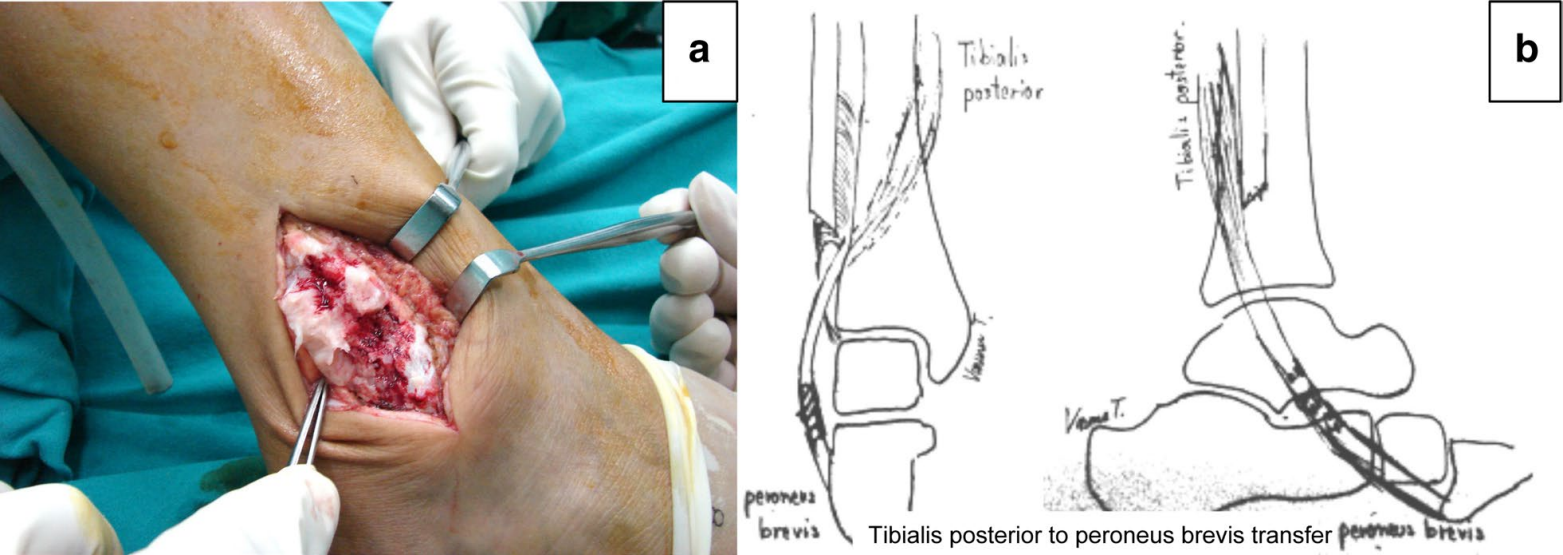
The direct lateral approach for definitive wide resection (**a**) and a diagram of tibialis posterior tendon transfer to the peroneus brevis for stabilization of the ankle (**b**)

The definitive surgeries included a wide resection of the right distal fibula and surrounding soft tissue on October 2011. The musculotendinous and tendinous part of the peroneus brevis and longus at the fibular bone were resected. After that, a medial incision at the navicular tuberosity was made. The tibialis posterior tendon was harvested from its insertion and retracted proximally. The tendon was passed under the tibia through the interossessous membrane. It was confirmed that the tunnel space was large enough for the tendon excursion without any kinking to the surrounding tissues. The level of the tunnel was at mid-tibia that allow tendon to transferred laterally by keeping the tendon axis in line. The tendon was sewn to the remaining distal part of the peroneus brevis with number 2 FiberWire. Tension of the ankle and subtalar joint were set in ankle neutral position (Fig. 3). The balance of lateral and medial stability maintained in position of ankle in neutral. Subcutaneous tissue and skin were closed layer by layer and a redivac drain was installed.

**Fig. 4:**
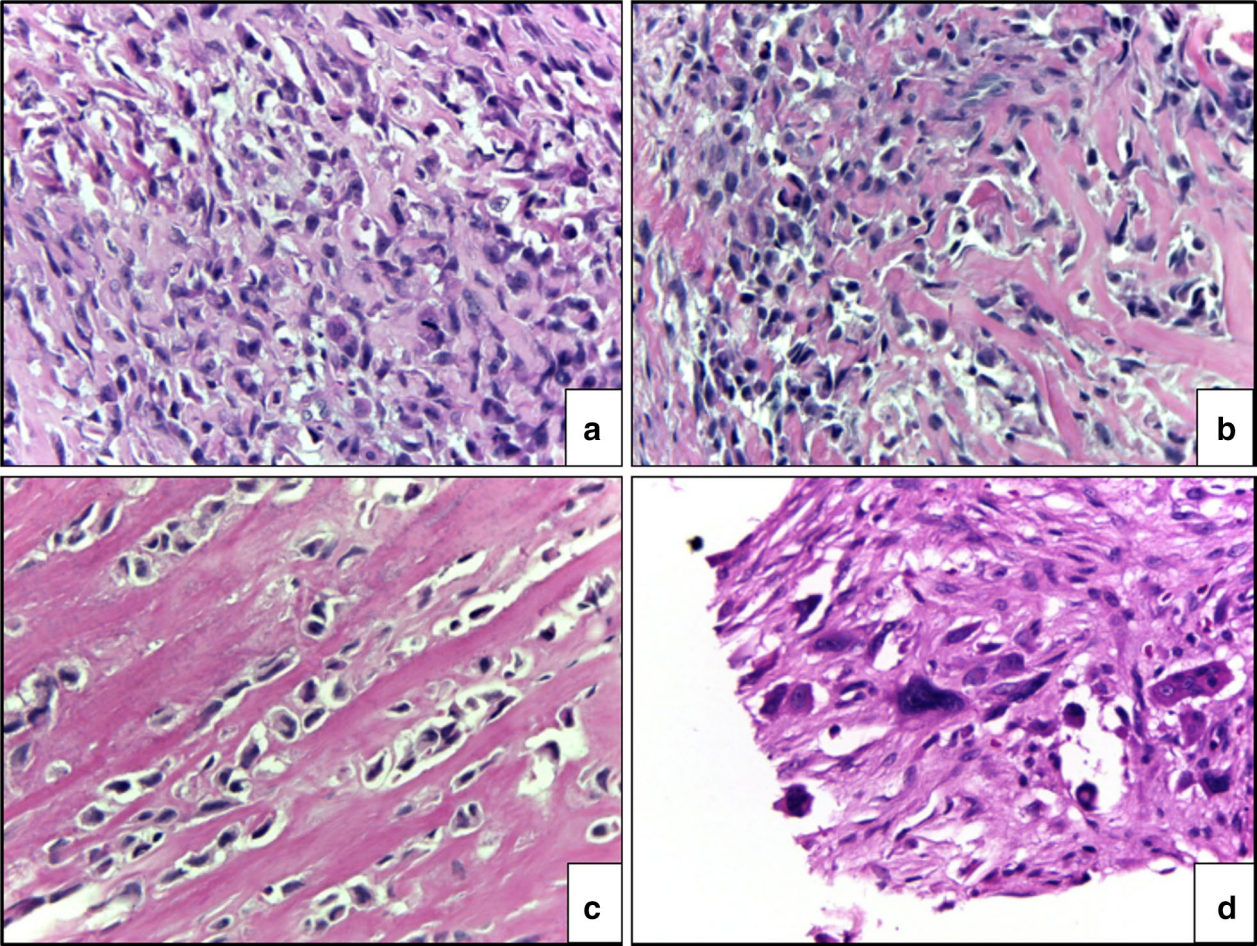
Histological results showing plump and spindle malignant cells with mitotic figures (magnification ×400, hematoxylin and eosin) (**a**), lace-like osteoid formation around the tumor cells (magnification ×400, hematoxylin and eosin) (**b**), thick neoplastic bone formation around the tumor cells (magnification ×400, hematoxylin and eosin) (**c**) and many large bizarre neoplastic cells in a post chemotherapy specimen (magnification ×400, hematoxylin and eosin) (**d**)

The definitive tissue pathology diagnosis was conducted on a sample consisting of a segment of distal fibula and surrounding soft tissue measuring 10.5 cm in length, 4 cm in width, and 3 cm in thickness. There was also a 10.5 cm × 1.5 cm piece of skin on the lateral side of the specimen. The bone had been previously cut longitudinally, showing a 2.5 cm × 1.5 cm white-tan hard solid tumor at the bony cortex and medulla. The tumor had broken through the bony cortex into the adjacent soft tissue. Histologically, there were numerous large size pleomorphic malignant cells (Fig. 4d) with less than 5% of the area showing tumor necrosis. Ongoing osteoid formation was observed.

**Fig. 5:**
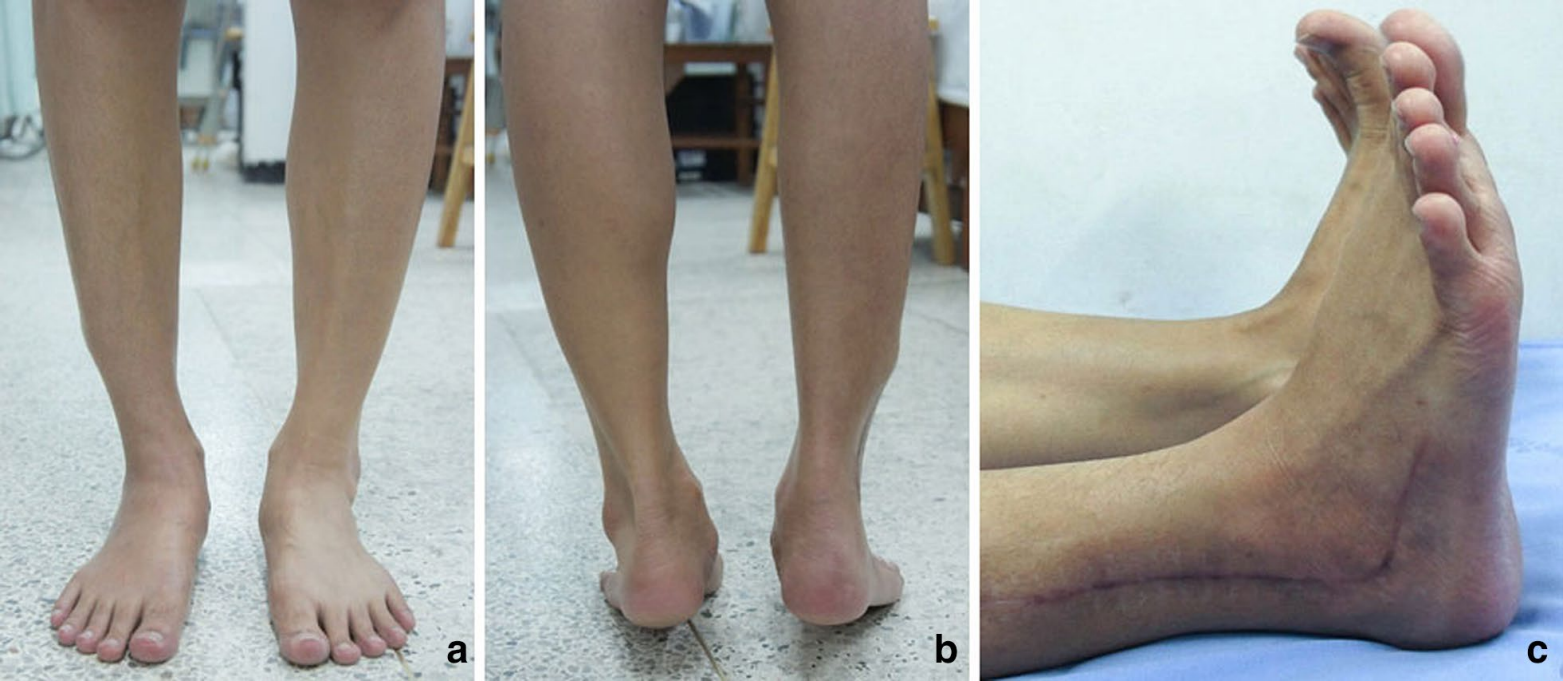
Clinical data at 24-months post-surgery showing stable ankle and functional outcomes (**a**, **b**) and the surgical scar (**c**)

The wound was completely healed within 2 weeks. Postoperative casting was applied for 2 months before commencing physical therapy. Chemotherapy with doxorubicin was continued for an additional 3 months after the operation. The symptom of pain was reduced and was completely relieved within 4 months. At 84 months after surgery, the patient could ambulate well with some limitations on daily activity. She walks with walking and dress shoes for most activity. She could stand on tiptoes. Moreover, she could do low-speed run, bicycle and jump. The plain radiographs showed no evidence of recurrent or metastatic cancer every year for 7 years (Fig. 5) The MRI scan of the ankle showed no evidence of recurrence at seventh year follow up.

## Discussion and conclusions

Malignant tumors of the lower extremities are uncommon. They have shown lower mortality rates then tumors in other sites. The fibula is involved in 2.4% of primary bone tumors, more often in the proximal one-third than the distal segment [5–7]. However, malignancies of the distal one-third of the fibula have a better prognosis than proximal lesions. The incidence of malignancy of the fibula in giant cell tumors and Ewing’s sarcomas is approximately 1 and 8%, respectively [8–10], while osteosarcomas of the fibula is approximately 2–5.6% of cases [2]. This case of osteosarcoma involved the distal one-third of the fibula without any metastatic lesion or soft tissue involvement.

Amputation, which in the past was the standard procedure for distal fibula tumors, has been replaced by wide resection made possible by more effective systemic treatments and surgical techniques [3]. Nevertheless, wide resection can be restricted by inadequate soft tissue coverage and by impacts on biomechanics of the foot and ankle. Moreover, that procedure can lead to a lack of stability requiring reconstruction of a stable joint and provision of sufficient skin coverage of the area [2, 11].

Different techniques of the distal fibula reconstruction after wide resection have been described. Capanna et al. reported the outcome of 11 benign or malignant tumors patients who underwent distal fibular resection by using different reconstruction techniques. At the final follow up, all patients were free of pain, 7 patients recovered normal function, while 4 patients presented reduced mobility and 1 patient developed lateral subluxation of the talus. Functional outcomes were acceptable [12].

Techniques of reconstruction promoting ankle stability with ankle arthrodesis or a prosthesis have been reported. Dieckmann et al. described the techniques of tibiotalocalcaneal fusion using a retrograde hindfoot nail or tibiotalar fusion with screws in a series of 11 distal fibular sarcomas or metastases patients who treated by resection of the entire fibula. This technique failed in 2 patients due to the cause of osteopenic bone [11]. Norman-Taylor et al. reported the outcomes of 5 patients with either Ewing’s sarcoma or osteosarcoma of the distal fibula treated by wide peroneal resections following with ankle arthrodesis. There were no local relapses in any of the cases. All patients were disease free at the time of follow-up [4]. In addition, Lee et al. described reconstruction with prostheses in 6 patients with aggressive benign or malignant bone tumors. Five of the tumors were in the distal tibia and one was in the distal fibula. The reconstruction was achieved using a custom-made, hinged prosthesis that replaced the distal tibia and the ankle. All patients had a good outcome and stable ankle joints. Complications occurred in two patients: one wound infection and one talar collapse [7].

The use of allograft or autograft to restore the lateral ankle stability of the bone has been found to have acceptable outcomes. Jamshidi et al. reported on 4 cases of distal fibular resection due to benign aggressive or malignant bone tumors and reconstruction with distal fibular allograft transplantation. A valgus deformity was found during follow-up in 1 case which was asymptomatic and no particularly treatment was given for the patient. All patients had nearly full ankle range of motion [13]. De Gauzy et al. described a case of 13-year-old boy with osteosarcoma of the distal fibula who underwent pedicled vascularized epiphyseal transfer using the ipsilateral proximal fibula. A good functional outcome and ankle stability were present at 2 years and 6 months [14].

Reconstruction techniques of the lateral ankle by ligament and tendon transfers have achieve good functional outcomes, but they are technically demanding. Sukimoto et al. reported a case of a 64-year-old patient who presented with the distal fibula metastasis treated with distal fibulectomy and the lateral ankle ligaments reconstruction using a patellar bone-tendon-bone allograft. At 1 year follow up, the patient’s ankle was pain free and had no instability [15]. Monson et al. presented the cases of 3 patients, 2 with Ewing sarcomas and 1 with a giant cell tumor. After distal fibular resection, reconstruction of the lateral ankle was done using the peroneus brevis tenodesis to the distal tibia and interwoven with the residual ankle ligaments. All patients were able to return to normal activities without presenting of ankle instability or early arthritis. However, there were complications of one sustained a traumatic fifth metatarsal base fracture that healed with conservative treatment and one distal fibular bursitis that underwent fibular shortening and bursectomy which achieved complete relief of the symptoms [16].

In our case report, the patient presented with a stable ankle and was pain-free 7 years after the distal fibula resection and ligament reconstruction. The advantages of this technique included no auto- or allograft implantation, no risk of nonunion from grafting, no donor site morbidity, and preservation of the mobility of the ankle joint. In addition, this technique could avoid tendon exposed or closed to the surgical skin incision at the foot if attachment of the tendon is on the midfoot bone such as lateral cuneiform. This technique is also adequate for balance setting of the hindfoot in neutral position without manipulation of the forefoot and midfoot. The disadvantages were a loss of bone contour and the need for a period of ambulation physical therapy. We recommended this ankle reconstructive procedure at the time of resection with the reasons of (1) avoiding of scar tissue during tendon reconstruction (2) avoiding ankle and subtalar joint arthrofibrosis that may occur in late stage procedure. It is important for a step of balancing medial and lateral stability of the hindfoot. The excellent functional score results at the mid-term follow-up confirm that ankle ligament reconstruction following distal fibula resection is an alternative treatment for malignant bone tumors that require wide resection of the bone and surrounding soft tissue. However, longer-term follow-up and a larger number of case series are needed to confirm these results.

In conclusion, the method of ankle ligament reconstruction after distal fibula and surrounding soft tissue resection described in this study is an option in cases where metal fixation or bone grafting are not required.

## Additional file

**Additional file 1.** Care checklist.

## Abbreviations

CT: computed tomography
GCTs: giant cell tumors
MRI: magnetic resonance, magnetic resonance imaging
