# Supplementary figures and images for: Ankle ligament reconstruction after wide resection of the osteosarcoma of the distal fibula: a case report
Source: BMC Res Notes. 2017 Dec 28;10:769. doi: 10.1186/s13104-017-3097-4 (PMC5808619; doi:10.1186/s13104-017-3097-4)

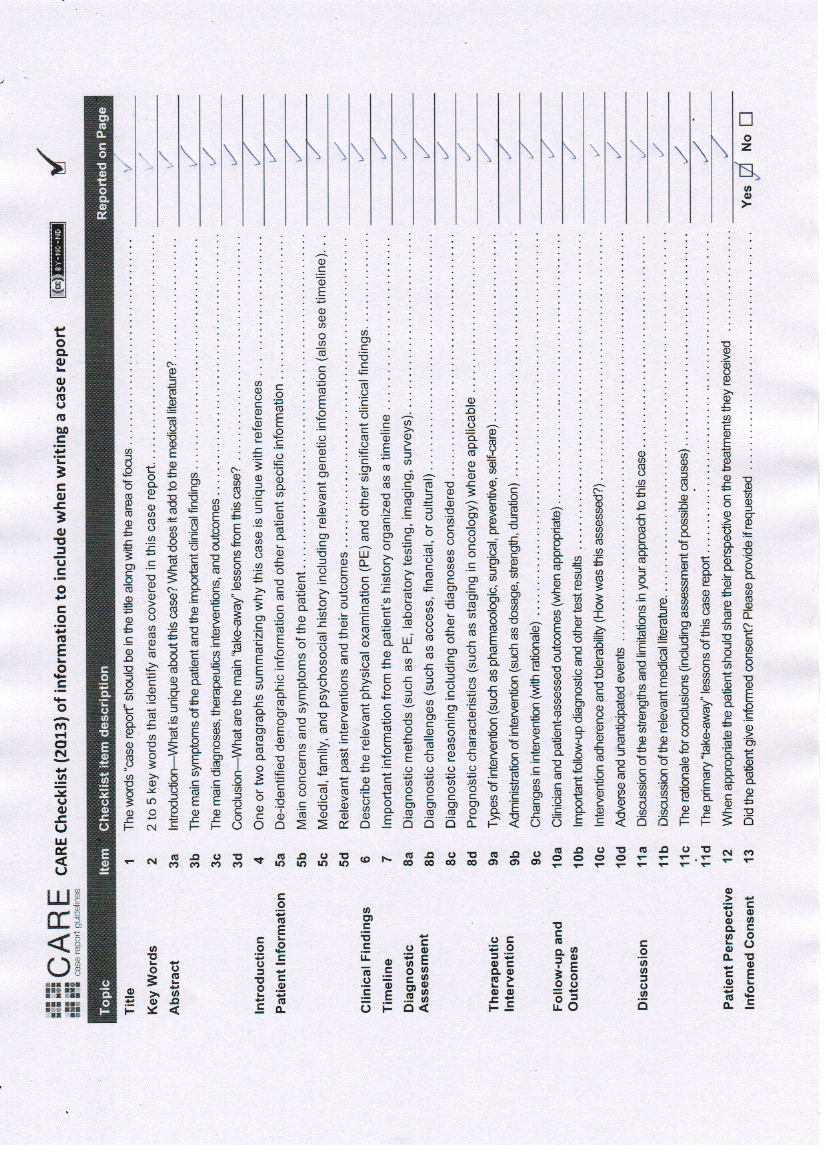

Supplement: Supplementary file 1 — Additional file 1. Care checklist. [file 13104_2017_3097_MOESM1_ESM.jpeg]
